# Crystallographic Studies Evidencing the High Energy Tolerance to Disrupting the Interface Disulfide Bond of Thioredoxin 1 from White Leg Shrimp *Litopenaeus vannamei*

**DOI:** 10.3390/molecules191221113

**Published:** 2014-12-15

**Authors:** Adam A. Campos-Acevedo, Enrique Rudiño-Piñera

**Affiliations:** Departamento de Medicina molecular y Bioprocesos, Instituto de Biotecnología (IBT), Universidad Nacional Autónoma de México (UNAM), Avenida Universidad 2001, Colonia Chamilpa, Cuernavaca 62210, Mexico

**Keywords:** disulfide bond, radiation damage, homodimer, thioredoxin 1, *Litopenaeus vannamei*

## Abstract

Thioredoxin (Trx) is a small 12-kDa redox protein that catalyzes the reduction of disulfide bonds in proteins from different biological systems. A recent study of the crystal structure of white leg shrimp thioredoxin 1 from *Litopenaeus vannamei* (*Lv*Trx) revealed a dimeric form of the protein mediated by a covalent link through a disulfide bond between Cys73 from each monomer. In the present study, X-ray-induced damage in the catalytic and the interface disulfide bond of *Lv*Trx was studied at atomic resolution at different transmission energies of 8% and 27%, 12.8 keV at 100 K in the beamline I-24 at Diamond Light Source. We found that at an absorbed dose of 32 MGy, the X-ray induces the cleavage of the disulfide bond of each catalytic site; however, the interface disulfide bond was cleaved at an X-ray adsorbed dose of 85 MGy; despite being the most solvent-exposed disulfide bond in *Lv*Trx (~50 Å^2^). This result clearly established that the interface disulfide bond is very stable and, therefore, less susceptible to being reduced by X-rays. In fact, these studies open the possibility of the existence in solution of a dimeric *Lv*Trx.

## 1. Introduction

In the X-ray diffraction of a protein crystal at an energy of 12.8 keV, about 98% of the incident photons pass through the crystal without interaction. Only 2% of ionizing energy interacts with the crystal. Of this 2%, about 1.84% corresponds to the photoelectric effect and inelastic scattering; only 0.16% corresponds to the elastic scattering responsible for the diffraction pattern phenomena [1]. Both the photoelectric effect and inelastic scattering cause the ionization of certain atoms in the crystal, causing chemical changes in the molecules that constitute the crystal and result in destabilization of the crystal lattice. 

The photoelectric effect makes it difficult to get good quality experimental data, since the order of the crystalline arrangement determines the properties of diffraction. Therefore, there is a compromise to obtain good parameters (higher resolution, I/(σI), completeness, redundancy, integrity, CC1/2, *etc.*), in order to minimize the effects caused by radiation damage during data collection. The X-rays induce site-specific changes in protein molecules, which include the reduction of metal ions and the breaking of certain covalent bonds [2,3], with subsequent disordering of the atoms forming the crystal lattice. 

The consequences of radiation damage are noticed in the covalent bond disruption [2,4,5], the decarboxylation of glutamic and aspartic residues [6,7], as well as the loss of hydroxyl groups of tyrosine residues and the methylthiol groups of methionine residues [4]. By extension from electron microscopy experiments at 77 K, a calculated dose of 20 MGy, the Henderson limit, was proposed as a limit in which the loss of half a power of diffraction in protein crystallography occurs [8]. Subsequently, the X-ray crystal’s absorbed dose was estimated, using protein crystals of holoferritin and apoferritin, a dose of 35 MGy, the Garman limit, in which the biological information coming from diffraction experiment may be compromised by radiation damage, was proposed [9]. 

With the development of third-generation synchrotrons and new X-ray free electron lasers (X-FEL), the X-ray absorbed dose in protein crystals exposed to those sources has increased from 10 to 1000 times as compared to home source X-rays, leading very frequently to final absorbed doses above 35 MGy. Most macromolecular crystals, which are kept at room temperature for data collection, lose their order after only a few seconds of exposure to the X-ray beams in a third-generation synchrotron. The use of cryogenic techniques has greatly reduced the radiation damage suffered during an experiment, allowing more exposure time to the X-ray without severely affecting the quality of diffraction. This is because the cryogenic temperature of 100 K restricts the diffusion of free radicals generated by radiation through the crystal lattice. Despite this tolerance, the effects of radiation damage continue to manifest themselves [10,11]. In studies conducted in a second-generation synchrotron using TcAChE (*Torpedo californica* acetyl cholinesterase), crystals under cryogenic conditions and HEWL (hen egg lysozyme) showed that the rupture of the disulfide bonds Cys254-Cys265 was produced in TcAChE and the Cys6-Cys127 disulfide bond in HEWL. Although these proteins have more disulfide bonds, these bonds were much more sensitive, because they are more accessible to the solvent than the others [2]. Besides this, variations in atomic displacement parameters (ADPs) for individual amino acids were analyzed. It was observed that the glutamic and aspartic residues showed an increase in ADP values in addition to the cysteines that mediated the disulfide bond formation. The increase in the ADPs may be a consequence of increased mobility or decarboxylation, an effect well known for ionizing radiation [2,12]. 

Although structural changes can occur in the crystal during the data collection, these changes are dependent on the crystal’s nature. The total radiation damage in cryopreserved protein crystals corresponds to the cumulative dose received during data collection. Recent reports have given a better understanding of the problem [13], and studies of radiation damage have reported that the damage is highly specific as the previously mentioned examples: TcAChE and HEWL [2,5]. In these cases, an integral study was done, revealing the existence of an increase in the ADPs, the increase in unit cell parameters, a slight rotation and translation of protein molecules, the disruption of disulfide bonds and the decarboxylation of aspartic and glutamic residues. All of these changes are initiated by the formation of free radicals during exposure to X-rays [2,5]. As a consequence of the formation of free radicals, the progressive disorder of the crystal and local chemical changes in the protein molecules is manifested. Further specific damage (loss of electron density) for the hydroxyl groups of tyrosine and the methylthiol group in methionine residues were also reported [1,4]. 

**Figure 1:**
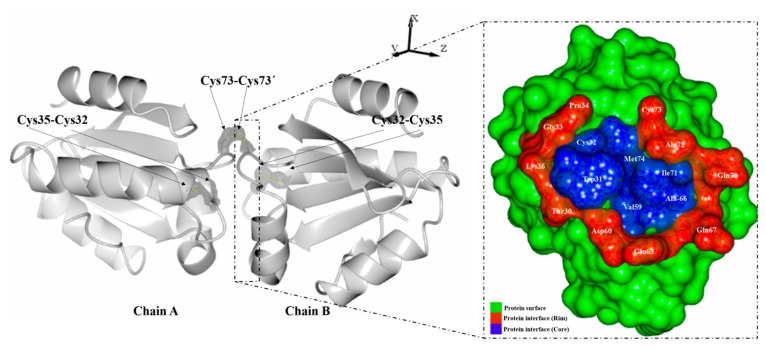
Crystallographic structure of *Lv*Trx, showing the dimer arrangement in the asymmetric unit for all deposited structures of *Lv*Trx in the PDB (3zzx, 4aj6, 4aj7, 4aj8). Electron-density maps 2Fo-Fc are shown in gray and contoured at 1.0 σ. The *Lv*Trx structure shows the location that has the interface disulfide bond (Cys73-Cys73'), which is the most exposed, compared with the catalytic disulfide bond of each monomer embedded in the dimeric interface. In the box with the dotted frame is shown the residues that are involved in the *Lv*Trx interface. The ratio of the interface is composed by the core in blue color (interface residues with at least one fully-buried atom) and the rim in red color (the other interface residues). The interface division by core/rim and the interface residues of *Lv*Trx were obtained by the program, ProFace [15]. The interface area was of 609.75 Å^2^ on average, determined by the PISA server (Protein Interfaces, Surfaces and Assemblies) at the European Bioinformatics Institute [16], and the figures were generated with CCP4mg.

*Litopenaeus vannamei* thioredoxin (*Lv*Trx) is a redox protein with a molecular weight of 12 kDa, which catalyzes the reduction of the disulfide bond in different biological systems. The determination of the crystal structures of *Lv*Trx under different redox conditions discloses a dimeric form mediated by a disulfide bond and other residues that are involved in the dimeric interface [14]. The contacts in the interface core area are formed by hydrophobic residues (Trp31, Val59, Ala66, Ile71 and Met74), blocking the access of water molecules. Other residues that compose the interface rim area of *Lv*Trx are mostly hydrophilic residues formed by Thr30, Cys32, Gly33, Pro34, Lys36, Asp60, Glu63, Gln67, Gln70, Ala72 and Cys73 (Figure 1).

In this study, we investigated the deterioration of disulfide bonds in two crystals of *Lv*Trx protein at different fluxes. The *Lv*Trx contains four cysteines; two of them are engaged to form a catalytic disulfide bond (Cys32 and Cys35); Cys62 is not involved in any interactions, but in human thioredoxin (hTrx), it has been reported that Cys62 is readily S-nitrosylated, giving a SNO modification [17]; and Cys73, which in previous studies, showed a dimeric behavior in the crystal system of hTrx and *Lv*Trx, mediated by the interface disulfide bond through the Cys73 residue of each monomer [14,17]. This conformation remained doubtful in *in vivo* experiments [18,19]. The importance of this study is focused on the interface disulfide bond of the *Lv*Trx, because this interaction remains stable, despite the presence of dithiothreitol (DTT) to a concentration of 5 mM, a sufficient concentration to maintain the catalytic disulfide bond in a reduced state during all of the crystallization process. 

In an attempt to determine if the interface disulfide bond is viable to be reduced or if it is a consequence of a crystalline arrangement, we used X-rays as a tool to conduct experiments of radiation damage to monitor the reduction of the interface disulfide bond in two crystals of *Lv*Trx. Each crystal was collected at 8% and 27% transmission energy, 1.33 × 10^11^ and 4.14 × 10^11^ photons s^−1^, respectively, with a series of 10 datasets collected per crystal in order to analyze the progressive reduction of the disulfide bonds and other changes.

These experiments shows the presence and reduction of the interface disulfide bond, from 3.4 MGy until reaching a dose of 85 MGy, showing the disulfide bond disruption and movement of the Cys73, Cys32 and Cys35 residues of each monomer. The interface disulfide bond is shown as one of the most stable disulfide bonds to our knowledge, even with a significant solvent exposure. This behavior opens the possibility to analyze those residues that are interacting at the interface area, due to these interactions being possibly crucial to maintain the interface bond stably and tolerably at high doses of energy, even when a reducing agents is added.

## 2. Results and Discussion

During data collection, two crystal of *Lv*Trx were diffracted at different transmissions, referred to below as *Lv*Trx-1x (8% of transmission, 1.33 × 10 ^11^ photons·s^−1^) and *Lv*Trx-3x (27% of transmission, 4.14 × 10^11^ photons·s^−1^). Ten datasets were collected for each crystal, with the purpose of analyzing the sequential increase in the absorbed dose of energy. Specifically, changes were observed in the electron density corresponding to the region of the interface disulfide bond (Cys73-Cys73') and the catalytic disulfide bond (Cys32-Cys35) of each monomer. The deterioration of the electron density was dependent on the increment of the absorbed dose. The calculation of absorbed dose was done with the program, RADDOSE [20,21,22].

In the case of the crystal *Lv*Trx-1x, the interface disulfide bond (Cys73-Cys73') remained stable, reaching a dose of 34 MGy. However, at a dose of 3.4 MGy, this disulfide bond showed double conformations in the Sγ atoms of the residues Cys73-Cys73' without compromising the disulfide bond formation. 

**Figure 2:**
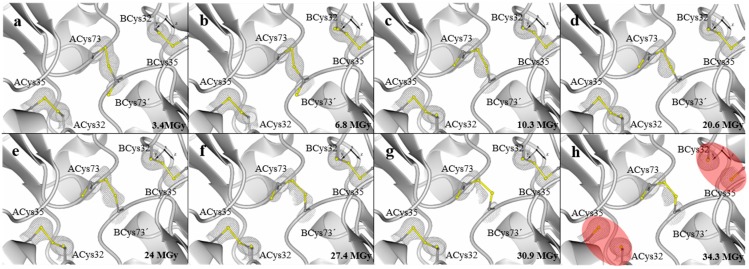
Crystal structures of *Lv*Trx-1x showing the disulfide bond deterioration (ACys32-ACys35, ACys73-BCys73' and BCys32-BCys35) caused by radiation damage in a sequential data collection of 10 datasets with 8% of transmission, 1.33 × 10^11^ photons s^−1^. Electron-density maps 2Fo-Fc are shown in gray and contoured at 1.0 σ. (**a**) First dataset collected at 3.4 MGy; (**b**) second dataset collected at 6.8 MGy; (**c**) third dataset collected at 10.3 MGy; (**d**) sixth dataset collected at 20.6 MGy; (**e**) seventh dataset collected at 24 MGy; (**f**) eighth dataset collected at 27.4 MGy; (**g**) ninth dataset collected at 30.9 MGy; (**h**) tenth dataset collected at 34.3 MGy, showing that up to this dose, the catalytic disulfide bond of chains A and B is fully reduced (highlighted in red ovals), while the interface disulfide bond remains stable. For practical purposes, the fourth and fifth datasets are not shown at a dose of 13.7 MGy and 17.1 MGy, respectively, since these datasets did not provide more information. The figures were generated with CCP4mg, and the video can be viewed in the Supplementary Material.

In the case of the catalytic disulfide bond (Cys32-Cys35) of each monomer, the dose at which the disulfide bond was fully reduced was at approximately 34 MGy (Figure 2a–h). We compared this behavior with other experiments focused on the radiation damage of disulfide bonds in different proteins. 

In the literature, it is reported that most of the covalent bonds were completely reduced by an absorbed dose of ~13 MGy or less. In protein crystals of lysozyme, all disulfide bonds were fully reduced at ~1 MGy (Cys6-Cys127; Cys30-Cys115; Cys64-Cys80; Cys76-Cys94) [23]; in protein crystals of TcAChE, only one of three disulfide bonds was fully reduced at an absorbed dose of ~6 MGy (Cys265-Cys254) [2]; in protein crystals of trypsin, only one of six disulfide bond was reduced at ~7 MGy (Cys191-Cys220) [24]; and in protein crystals of elastase, all disulfide bonds were fully reduced at ~13 MGy (Cys58-Cys42; Cys182-Cys-168; Cys220-Cys191; Cys136-Cys201) [7] (Table S1). 

It is important to mention that the catalytic site of all the Trxs is very reactive and dynamic, with different targets [25]. Therefore, the determined absorbed dose at which the disruption of the catalytic disulfide bond occurs in *Lv*Trx seems high.

For the crystal *Lv*Trx-1x, the absorbed dose calculated is on the Garman limit reported by Owen *et al.*, 2006, at 35 MGy [9], showing an example of biological information not ever affected above the Garman limit. 

One possible explanation whereby the catalytic disulfide bond is stable at a high absorbed dose of energy is due to the dimer arrangement of the *Lv*Trx in the crystal lattice (Figure 1). The catalytic disulfide bond of each monomer is embedded in the dimer interface area, thereby causing a tolerance to radiation damage, the solvent-accessible surface area (ASA) of each of the cysteines of ACys32: 0 A^2^, ACys35: 0.2 Å^2^ and BCys32: 0 Å^2^, BCys35: 4.2 Å^2^ being calculated by AREAIMOL [26,27]. In Table S2, the disulfide bonds’ ASA of the *Lv*Trx are comparable with other monomeric Trxs.

**Figure 3:**
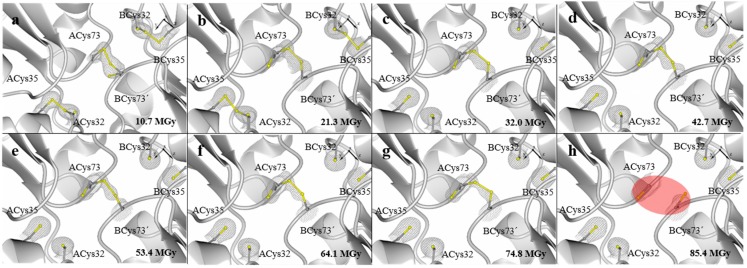
Crystal structures of *Lv*Trx-3x showing the deterioration of the catalytic disulfide bond (ACys32-ACys35and BCys32-BCys35) and the interface disulfide bond (Cys73-Cys73') caused by radiation damage. The energy with which each dataset was obtained of the crystal *Lv*Trx-3x was three-times higher (27% transmission, 4.14 × 10^11^ photons s^−1^) than the crystal *Lv*Trx-1x. (**a**) First dataset collected at 10.7 MGy; (**b**) second dataset collected at 21.3 MGy; (**c**) third dataset collected at 32 MGy, a dose at which the catalytic disulfide bond is completely reduced, similar to what was obtained in the crystal *Lv*Trx-1x; (**d**) fourth dataset collected at 42.7 MGy; (**e**) fifth dataset collected at 53.4 MGy; (**f**) Sixth dataset collected at 64.1 MGy; (**g**) seventh dataset collected at 74.8 MGy; (**h**) eighth dataset collected at 85.4 MGy, showing that at this dose, the interface disulfide bond is completely broken (highlighted in red oval). Datasets 9 and 10 are not shown, because the expected result was obtained in Dataset 8. Electron-density maps 2Fo-Fc are shown in gray and contoured at 1.0 σ. The figures were generated with CCP4mg, and the video can be viewed in the Supplementary Material.

In the case of the crystal of *Lv*Trx-3x, the structure analysis showed that the catalytic disulfide bond was disrupted at a dose of 32 MGy. Similar data were calculated for the crystal *Lv*Trx-1x, when the catalytic disulfide bond was broken. Despite the fact that cysteine mediated the interface disulfide bond, this residue exhibits a double conformation in the seven datasets. The disulfide bond was stable up to a dose of 85 MGy, in which the reduction was complete (Figure 3a–h).

The tolerance that the interface disulfide bond has to radiation damage is unusual, because this disulfide bond is exposed to the solvent with an ASA of ~50 Å^2^ (Table S2). This result suggests that the maintenance of this covalent interaction could depend on the residues involved in the dimer interface of the *Lv*Trx. This residue interaction at the interface area is mostly hydrophobic and could be limiting the flexibility of this disulfide bond, justifying the high tolerance to radiation damage. The summary of sequential deterioration of the catalytic and the interface disulfide bond is shown in Figure S1, and the video can be viewed in the Supplementary Materials.

## 3. Experimental Section

### 3.1. Experimental Procedures

The recombinant *Lv*Trx was purified in a similar manner as in previously published studies [14]. Two crystals of white leg shrimp *Litopenaeus vannamei* thioredoxin (*Lv*Trx-3x and *Lv*Trx-1x) were independently grown using the same crystallization conditions. Both crystals were grown from 0.1 M sodium acetate trihydrate, pH 4.6, 2.0 M ammonium sulfate with a protein concentration of 6 mg mL^−1^ and cryo-cooling with 30% (*v*/*v*) glycerol in liquid nitrogen, as previously described by Campos-Acevedo *et al.*, 2013 [14]. The approximate dimensions for the crystal *Lv*Trx-1x were 0.2 × 0.04 × 0.04 mm and 0.3 × 0.06 × 0.06 mm for the crystal *Lv*Trx-3x. In this study, 10 datasets of each crystal were collected at 100 K in the beamline I-24 (λ = 0.97 Å, 12.8 keV) at the Diamond Light Source (DLS) in Oxford, UK. 

### 3.2. Data Collection and X-ray Diffraction Experiments

The goal of the experiment was to monitor the overall and local changes that occur in the crystal at increasing dosages. During the experiment, ten datasets (Datasets 1–10) were collected consecutively from a single region of each crystal at 100 K. The crystal orientation, the rotation range (0.2°), the exposure time per image (0.2 s), the number of images collected per dataset (300 images), the wavelength (0.97 Å) and the crystal to detector distance (300 mm) were the same for all datasets, except for the percentage of transmittance (fluxes of 1.33 × 10^11^ and 4.14 × 10^11^ photons·s^−1^) for *Lv*Trx-1x and *Lv*Trx-3x crystals, respectively. The crystal was oriented with the longest dimension along the spindle. The slit size was 0.03 × 0.03 mm horizontal × vertical. The dose absorbed by the crystal for each dataset was calculated using the program, RADDOSE [20,21,22].

### 3.3. Data Processing, Molecular Replacement and Refinement

For all of the recombinant *Lv*Trx, datasets were determined by the molecular replacement method (MR). The initial phases were obtained using the full-length thioredoxin from the *Litopenaeus vannamei* structure previously deposited [14], using PDB Code 3zzx as a search model. The diffraction images were integrated using XDS [28], and the scaling was performed with SCALA from the CCP4 suite (Collaborative Computational Project, Number 4) [27]. The crystal belonged to space group p3_2_12, with unit cell parameters a = 57.7 ± 0.4 Å, b = 57.7 ± 0.4 Å, c = 118 ± 0.8 Å; α = 90°, β = 90°, γ = 120°. Additionally, Pointless [29] clearly supported the space group selection for each structure determination. A cross-rotational search followed by a translational search was performed using the program, Phaser [30], to obtain an initial model and the phases. The model was improved based on manual inspection of the 2Fo-Fc map after a rigid-body refinement and geometric constraint were performed in REFMAC [31]. All further refinements were performed using the program, PHENIX [32]. The final model was completed using PHENIX and Coot [33]. Data-collection statistics are summarized in Table 1 and Table 2.

**Table 1:** Summary of crystallographic data of *Lv*Trx-1x. Values in parentheses are for the highest resolution shell.

Parameters	*Lv*Trx-1x (1)	*Lv*Trx-1x (2)	*Lv*Trx-1x (3)	*Lv*Trx-1x (6)	*Lv*Trx-1x (7)	*Lv*Trx-1x (8)	*Lv*Trx-1x (9)	*Lv*Trx-1x (10)
**Data Collection Statistics**								
X-ray source	DLS Beamline I24	DLS Beamline I24	DLS Beamline I24	DLS Beamline I24	DLS Beamline I24	DLS Beamline I24	DLS Beamline I24	DLS Beamline I24
Wavelength (Å)	0.9686	0.9686	0.9686	0.9686	0.9686	0.9686	0.9686	0.9686
Space group	P3_2_12	P3_2_12	P3_2_12	P3_2_12	P3_2_12	P3_2_12	P3_2_12	P3_2_12
Absorbed dose (MGy)	3.4	6.8	10.3	20.6	24.0	27.4	30.9	34.3
Unit-cell dimensions								
*a, b, c* (Å)	57.18, 57.18, 117.62	57.21, 57.21, 117.75	57.24, 57.24, 117.85	57.44, 57.44, 117.90	57.41, 57.41, 117.85	57.36, 57.36, 117.85	57.46, 57.46, 117.90	57.21, 57.21, 117.95
α, β, γ angles (°)	90.0, 90.0, 120.0	90.0, 90.0, 120.0	90.0, 90.0, 120.0	90.0, 90.0, 120.0	90.0, 90.0, 120.0	90.0, 90.0, 120.0	90.0, 90.0, 120.0	90.0, 90.0, 120.0
Resolution range (Å)	28.59–1.65	28.60–1.71	28.61–1.65	45.83–1.72	45.80–1.74	45.75–1.76	45.85–1.80	27.80–1.84
No. of reflections	86,984	76,685	84,052	63,098	61,524	61,048	61,404	61,761
No. of unique reflections	26,586	23,647	25,952	23,101	22,342	21,661	20,239	18,958
Completeness (%)	99.9 (99.8)	98.0 (91.6)	98.2 (92.8)	96.7 (95.5)	97.0 (96.1)	97.3 (95.7)	96.6 (96.4)	97.3 (90.7)
*R_svm_* (%) ^§^	3.8 (43.9)	3.4 (34.3)	3.7 (50.5)	6.9 (39.1)	6.9 (39.6)	7.6 (40)	5.2 (40.4)	4.8 (42.4)
*I/*σ*(I)*	15.9 (2.4)	18.0 (2.9)	15.0 (2.0)	7.5 (2.0)	7.7 (2.0)	7.3 (2.0)	7.2 (1.36)	13.0 (2.8)
Multiplicity	3.3 (3.3)	3.2 (3.0)	3.2 (3.0)	2.7 (2.5)	2.8 (2.6)	2.8 (2.6)	2.5 (2.2)	3.3 (3.2)
Asymmetric unit	Dimer	Dimer	Dimer	Dimer	Dimer	Dimer	Dimer	Dimer
**Refinement Statistics**								
*R_work_/R_free_* (%)	18.87/22.33	18.39/23.41	19.22/23.15	18.77/22.98	18.76/23.72	18.66/23.93	18.66/23.37	18.45/21.72
B-value (Å^2^)								
Protein	25.2	26.8	30.4	25.53	26.31	25.05	25.64	28.52
Ion/Ligand	32.21	35.63	38.31	32.69	34.35	35.97	35.47	51.61
Water	37.69	38.98	41.64	37.68	37.46	36.74	36.75	39.21
All atoms	31.63	33.80	36.78	31.96	32.70	32.58	32.62	39.78
Wilson plot B-value (Å^2^)	24.97	25.8	26.46	25.93	26.67	27.21	27.90	30.01
**RMSD from Ideal Stereochemistry**								
Bond lengths (Å)	0.005	0.005	0.005	0.005	0.005	0.004	0.005	0.005
Bond angles (°)	0.924	0.899	0.921	0.940	0.932	0.816	0.867	0.885
Coordinate error (maximum-likelihood base) (Å)	0.19	0.23	0.20	0.26	0.25	0.26	0.24	0.21
Ramachandran plot (%)								
Most-favored regions	97.75	97.78	97.32	97.32	97.77	97.32	97.31	97.75
Additional allowed regions	2.25	2.22	2.68	2.68	2.23	2.68	2.69	2.25
Disallowed regions	0	0	0	0	0	0	0	0

^§^ R*_sym_* = Ʃ*_hkl_* Ʃ*_i_* |*I_i_(hkl)* − *(I(hkl)*|Ʃ*_hkl_* Ʃ*_i_ I_i_ (hkl)*, where *I_i_(hkl)* and *(I(hkl))* represent the diffraction-intensity values of the individual measurements and the corresponding mean values. The summation is over all unique measurements.

**Table 2:** Summary of crystallographic data of *Lv*Trx-3x. Values in parentheses are for the highest resolution shell.

Parameters	*Lv*Trx-3x (1)	*Lv*Trx-3x (2)	*Lv*Trx-3x (3)	*Lv*Trx-3x (4)	*Lv*Trx-3x (5)	*Lv*Trx-3x (6)	*Lv*Trx-3x (7)	*Lv*Trx-3x (8)
**Data Collection Statistics**								
X-ray source	DLS Beamline I24	DLS Beamline I24	DLS Beamline I24	DLS Beamline I24	DLS Beamline I24	DLS Beamline I24	DLS Beamline I24	DLS Beamline I24
Wavelength (Å)	0.9686	0.9686	0.9686	0.9686	0.9686	0.9686	0.9686	0.9686
Space group	P3_2_12	P3_2_12	P3_2_12	P3_2_12	P3_2_12	P3_2_12	P3_2_12	P3_2_12
Absorbed dose (MGy)	10.7	21.3	32.0	42.7	53.4	64.1	74.8	85.4
Unit-cell dimensions								
*a, b, c* (Å)	57.49, 57.49, 117.92	57.55, 57.55, 118.02	57.61, 57.61, 118.05	57.65, 57.65, 118.06	57.68, 57.68, 118.05	57.70, 57.70, 118.05	57.69, 57.69, 118.02	57.70, 57.70, 118.02
α, β, γ angles (°)	90.0, 90.0, 120.0	90.0, 90.0, 120.0	90.0, 90.0, 120.0	90.0, 90.0, 120.0	90.0, 90.0, 120.0	90.0, 90.0, 120.0	90.0, 90.0, 120.0	90.0, 90.0, 120.0
Resolution range (Å)	28.75–1.46	27.95–1.60	28.80–1.70	25.90–1.80	28.01–1.88	28.02–1.96	28.02–2.05	28.85–2.15
No. of reflections	108,975	97,790	81,543	69,057	60,539	53,701	46,718	40,484
No. of unique reflections	38,937	29,741	24,912	21,055	18,546	16,387	14,356	12,470
Completeness (%)	99.6 (99.4)	99.6 (99.4)	99.6 (99.6)	99.8 (99.6)	99.8 (99.6)	99.9 (99.6)	99.6 (99.7)	99.6 (99.6)
*R_sym_*(%) ^§^	2.5 (38.4)	2.7 (39.9)	2.9 (44.0)	3.3 (40.5)	3.9 (42.1)	4.5 (45)	4.8 (42.6)	5.0 (36.7)
*I/*σ*(I)*	21.8 (3.2)	21.5 (3.1)	21.1 (2.8)	19.2 (3.1)	16.7 (2.9)	14.9 (2.9)	14.4 (3.0)	14.1 (3.5)
Multiplicity	3.3 (3.2)	3.3 (3.3)	3.3 (3.2)	3.3 (3.3)	3.3 (3.2)	3.3 (3.2)	3.3 (3.3)	3.2 (3.2)
Asymmetric unit	Dimer	Dimer	Dimer	Dimer	Dimer	Dimer	Dimer	Dimer
**Refinement Statistics**								
*R_work_/R_free_*(%)	19.95/22.02	18.67/23.36	18.71/20.67	18.58/22.65	18.23/22.63	17.78/21.72	18.66/23.37	18.03/24.63
B-value (Å^2^)								
Protein	24.83	23.88	28.13	29.22	31.2	34.54	34.29	36.59
Ion/Ligand	47.50	42.28	52.88	51.69	55.57	61.46	52.27	64.22
Water	38.0	36.65	40.39	41.02	41.61	43.77	42.05	44.55
All atoms	36.77	34.27	40.46	40.64	42.79	46.65	42.87	48.45
Wilson plot B-value (Å^2^)	20.55	23.61	26.69	29.43	31.99	34.28	36.30	37.78
**RMSD from Ideal Stereochemistry**								
Bond lengths (Å)	0.004	0.006	0.004	0.004	0.005	0.004	0.004	0.006
Bond angles (°)	0.861	1.027	0.824	0.826	0.932	0.842	0.756	0.938
Coordinate error (maximum-likelihood base) (Å)	0.18	0.18	0.17	0.21	0.23	0.19	0.24	0.25
Ramachandran plot (%)								
Most-favored regions	97.77	97.32	97.75	97.30	98.20	97.75	98.19	97.75
Additional allowed regions	2.23	2.68	2.25	2.70	1.80	2.25	1.81	2.25
Disallowed regions	0	0	0	0	0	0	0	0

^§^ R*_sym_* = Ʃ*_hkl_* Ʃ*_i_* |*I_i_(hkl) − (I(hkl)*| Ʃ*_hkl_* Ʃ*_i_ I_i_ (hkl)*, where *I_i_(hkl)* and *(I(hkl))* represent the diffraction-intensity values of the individual measurements and the corresponding mean values. The summation is over all unique measurements.

## 4. Conclusions

The analysis of our experiments with two crystals of *Lv*Trx show a specific radiation damage in the catalytic and the interface disulfide bond as a result of the sequential increase in the absorbed dose during the collection of each dataset. This analysis shows that the catalytic disulfide bond deterioration is less tolerant to radiation damage than the disulfide bond located in the dimeric interface with a calculated absorbed dose of 32 MGy and 85 MGy, respectively. In the case of the catalytic and the interface disulfide bonds of *Lv*Trx, the disruption of the *Lv*Trx catalytic disulfide bond is at least two-fold more tolerant in comparison to previous experiments of radiation damage focusing on the disulfide bond reduction (32 MGy *vs*. ~ 13 MGy), even while having a compromising solvent accessible area.

The tolerance to high doses of radiation by the catalytic disulfide bond in *Lv*Trx may be due to its poor accessibility to the solvent as a result of *Lv*Trx dimer arrangement in the crystalline state. In the case of the interface disulfide bond, it was most exposed to the solvent, which theoretically would make it the most susceptible to radiation damage; however, the disulfide bond remains intact at high energy dosages. The interaction that maintains the dimer interface not only depends on a covalent bond, but also on the other residues that compound the interface of each monomer [14]. This protein-protein interaction plays a crucial role in the stability of the interface disulfide bond, thereby limiting the flexibility of the structure and compromising the reduction of the disulfide bond. 

The disruption of the interface disulfide bond opens up the possibility that the dimeric form in *Lv*Trx is also possible in solution. Although several authors previously reported this dimeric arrangement in a Trx crystal system [14,17,34,35], there still remains no evidence of this behavior in solution. It would be interesting to see if the *Lv*Trx protein can be used as a model to study this behavior to know if the dimerization present in the crystal lattices is the same in solution and if the oligomeric state has any physiological role.

## Supplementary Materials

Supplementary materials can be accessed at: http://www.mdpi.com/1420-3049/19/12/21113/s1.
