# Supplementary material for: Crystallographic Studies Evidencing the High Energy Tolerance to Disrupting the Interface Disulfide Bond of Thioredoxin 1 from White Leg Shrimp Litopenaeus vannamei
Source: Molecules. 2014 Dec 15;19(12):21113–26. doi: 10.3390/molecules191221113 (PMC6270739; doi:10.3390/molecules191221113)
Supplement: Supplementary file 1 [file molecules-19-21113-s001.zip › molecules-67560-supplementary video.pptx]

## Slide 1
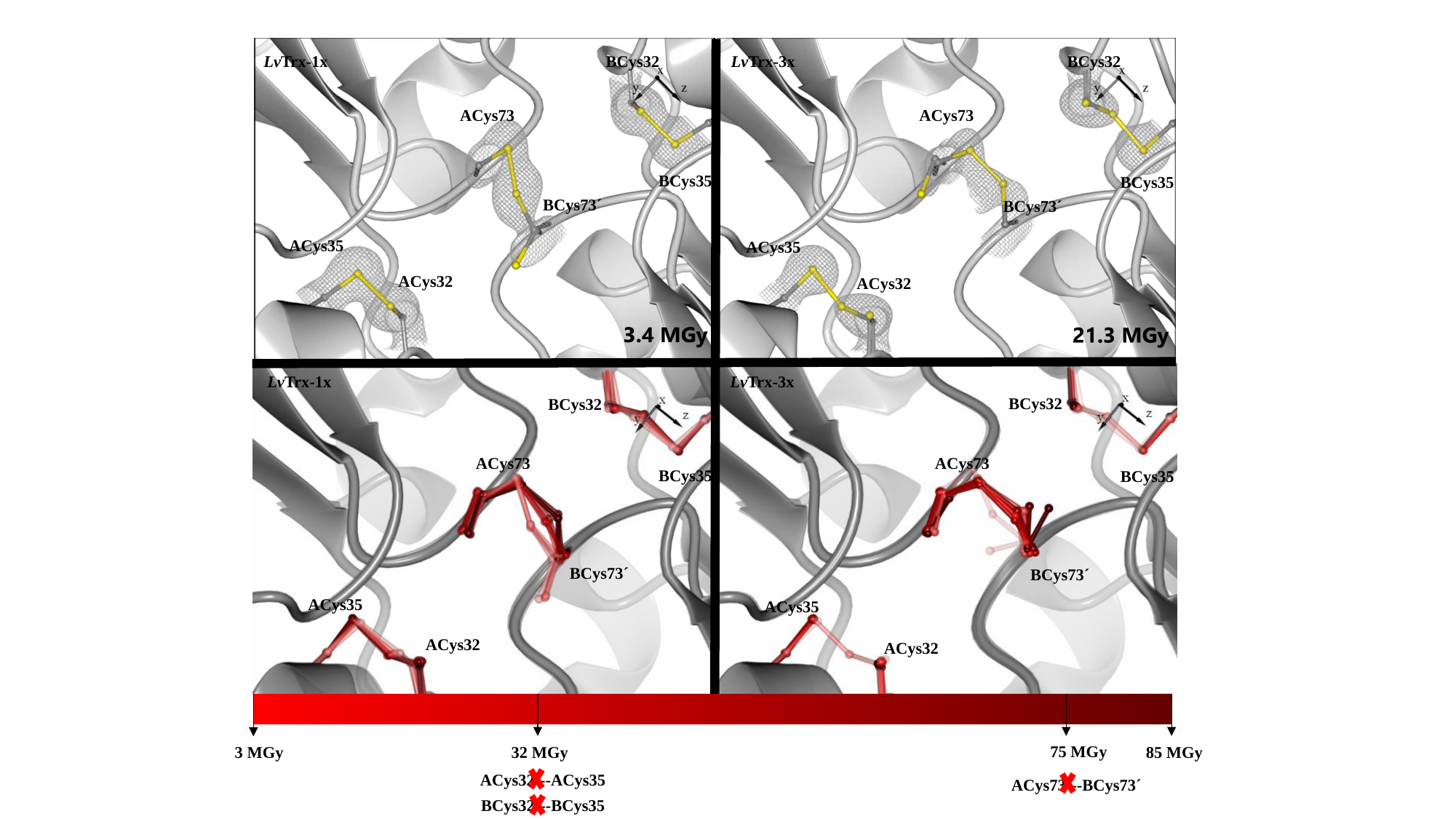

BCys32
ACys73
BCys35
BCys73´
ACys35
ACys32
LvTrx-3x
BCys32
ACys73
BCys35
BCys73´
ACys35
ACys32
LvTrx-1x
BCys32
ACys73
BCys35
BCys73´
ACys35
ACys32
BCys32
ACys73
BCys35
BCys73´
ACys35
ACys32
LvTrx-1x
LvTrx-3x
75 MGy
3 MGy
32 MGy
85 MGy
ACys32---ACys35
ACys73---BCys73´
BCys32---BCys35
