# Supplementary material for: Crystallographic Studies Evidencing the High Energy Tolerance to Disrupting the Interface Disulfide Bond of Thioredoxin 1 from White Leg Shrimp Litopenaeus vannamei
Source: Molecules. 2014 Dec 15;19(12):21113–26. doi: 10.3390/molecules191221113 (PMC6270739; doi:10.3390/molecules191221113)

Supplementary Materials

Table S1. Specific radiation damage to the disulfide bridges of different proteins as well as the area of solvent exposure for each cysteine. The absorbed dose is given in MGy. In red color is underlined those disulfide bonds that were reduced by radiation damage experiments.

Disulfide Bond	Residue	Solvent Accessibility (Å^2)	Absorbed Dose in MGy
		**Lisozyme**	
Cys6-Cys127	Cys6	43	1 MGy<br>PDB 4h8x, 4h8y, 4h8z,4h90, 4h91, 4h92, 4h93, 4h94, 4h9a, 4h9b, 4h9c, 4h9e, 4h9f, 4h9h, 4h9i
	Cys127	21	
Cys30-Cys115	Cys30	0	
	Cys115	0	
Cys64-Cys80	Cys64	0	
	Cys80	1	
Cys76-Cys94	Cys76	20	
	Cys94	2	
		**TcAChE**	
Cys67-Cys94	Cys67	11.2	6 MGy<br>PDB 1qid, 1qie, 1qif, 1qig, 1qih, 1qii, 1qij, 1qik, 1qim
	Cys94	3.5	
Cys254-Cys265	Cys254	18.8	
	Cys265	20.4	
Cys402-Cys521	Cys402	2.1	
	Cys521	11.9	
		**Elastase**	
Cys58-Cys42	Cys58	4.2	13 MGy<br>PDB 3mnb, 3mnc, 3mns, 3mnx, 3mo3, 3mo6, 3mo9, 3moc, 3mty, 3odf, 3mu0, 3mu1, 3mu4, 3odd, 3mu5, 3mu8
	Cys42	1.3	
Cys182-Cys168	Cys182	3.2	
	Cys168	0	
Cys220-Cys191	Cys220	1.8	
	Cys191	5.4	
Cys136-Cys201	Cys136	0.1	
	Cys201	0	
		**Trypsin**	
Cys157-Cys22	Cys157	2.3	7 MGy<br>PDB 1hj8, 1hj9
	Cys22	21.5	
Cys58-Cys42	Cys58	3.1	
	Cys42	4.0	
Cys232-Cys128	Cys232	24.7	
	Cys128	68.6	
Cys201-Cys136	Cys201	2.2	
	Cys136	1.2	
Cys168-Cys182	Cys168	0	
	Cys182	0	
Cys191-Cys220	Cys191	2.4	
	Cys220	11.4	
		**Thioredoxin *Lv*Trx**	
Cys73-Cys73'	Cys73	23.3	0.67 MGy<br>PDB 4aj7, 4aj8, 3zzx, 4aj6
	Cys73'	36.5	
ACys32-ACys35	ACys32	0	
	ACys35	0.2	
BCys32-BCys35	BCys32	0	
	BCys35	4.2	

Table S2. Comparison of solvent accessibility by the catalytic disulfide and Trx interface in different species depending on the arrangement in the crystal lattice (dimer and monomer).

Disulfide Bond	Residue	Solvent Accessibility (Å^2)	Absorbed Dose in MGy
**Thioredoxin *Lv*Trx Dimer**			
Cys73-Cys73'	Cys73	23.3	0.013 MGy<br>PDB 3zzx<br>Catalytic site<br>Partially reduced
	Cys73'	36.5	
ACys32-ACys35	ACys32	0	
	ACys35	0.2	
BCys32-BCys35	BCys32	0	
	BCys35	4.2	
**Thioredoxin LvTrx Dimer**			
Cys73-Cys73'	Cys73	27.5	0.51 MGy<br>PDB 4aj6<br>Catalytic site reduced
	Cys73'	32.1	
ACys32-ACys35	ACys32	0	
	ACys35	5.4	
BCys32-BCys35	BCys32	0	
	BCys35	4.7	
**Thioredoxin LvTrx Dimer**			
Cys73-Cys73'	Cys73	20.7	0.67 MGy<br>PDB 4aj7<br>Catalytic site oxidized
	Cys73'	38.8	
ACys32-ACys35	ACys32	0	
	ACys35	4.4	
BCys32-BCys35	BCys32	0	
	BCys35	3.7	
**Thioredoxin LvTrx Dimer**			
Cys73-Cys73'	Cys73	27.7	0.019 MGy<br>PDB 4aj8<br>Catalytic site<br>Partially reduced
	Cys73'	28.3	
ACys32-ACys35	ACys32	0	
	ACys35	0	
BCys32-BCys35	BCys32	0	
	BCys35	0	
**Human Thioredoxin Monomer**			
Cys73	Cys73	62.2	Unkown<br>1ert<br>Catalytic site reduced
ACys32-ACys35	ACys32	14.5	
	ACys35	6.9	
**Human Thioredoxin Monomer**			
Cys73	Cys73	65.6	Unkown<br>1eru<br>Catalytic site Oxidized
ACys32-ACys35	ACys32	6.3	
	ACys35	1.9	
***E. coli* Thioredoxin Monomer**			
ACys32-ACys35	ACys32	5.2	Unkown<br>2trx<br>Catalytic site Oxidized
	ACys35	3.3	
***D. melanogaster* Thioredoxin Monomer**			
ACys32-ACys35	ACys32	6.9	Unkown<br>1xw9<br>Catalytic site Oxidized
	ACys35	2.8	

Figure S1. Sequential deterioration of the catalytic and the interface disulfide bond at continue X-ray exposure. In the top panel are shown the first images of videos to correspond to crystal *Lv*Trx-1x and *Lv*Trx-3x. The videos can be visualized in an external file. In the below panel, the summary of the catalytic and interface disulfide bonds changes are shown during exposure to X-ray. Colors from white to deep red illustrate the dose received during the data collection. The black arrows indicate the dose in which these disulfide bonds are broken.

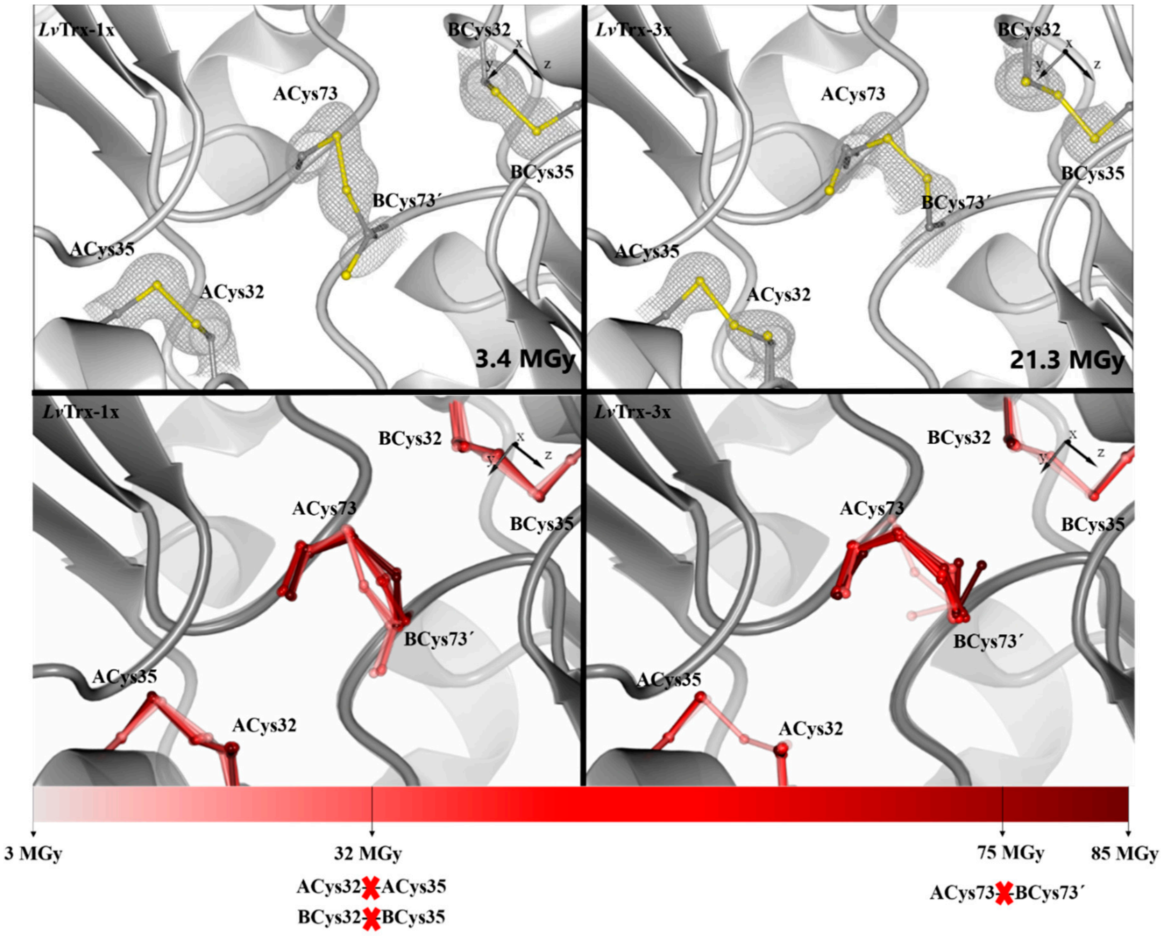

Supplement: Supplementary file 1 [file molecules-19-21113-s001.zip › molecules-67560-Supplementary materials.pdf]
